# AlphaFold2 Reveals Structural Patterns of Seasonal Haplotype Diversification in SARS-CoV-2 Nucleocapsid Protein Variants

**DOI:** 10.3390/v16091358

**Published:** 2024-08-25

**Authors:** Muhammad Asif Ali, Gustavo Caetano-Anollés

**Affiliations:** Evolutionary Bioinformatics Laboratory, Department of Crop Sciences, University of Illinois at Urbana-Champaign, Urbana, IL 61801, USA; maa32@illinois.edu

**Keywords:** COVID-19, haplotypes, nucleocapsid protein, mutation, pandemic, protein structure, recruitment, variant of concern, virus evolution

## Abstract

The COVID-19 pandemic saw the emergence of various Variants of Concern (VOCs) that took the world by storm, often replacing the ones that preceded them. The characteristic mutant constellations of these VOCs increased viral transmissibility and infectivity. Their origin and evolution remain puzzling. With the help of data mining efforts and the GISAID database, a chronology of 22 haplotypes described viral evolution up until 23 July 2023. Since the three-dimensional atomic structures of proteins corresponding to the identified haplotypes are not available, ab initio methods were here utilized. Regions of intrinsic disorder proved to be important for viral evolution, as evidenced by the targeted change to the nucleocapsid (N) protein at the sequence, structure, and biochemical levels. The linker region of the N-protein, which binds to the RNA genome and self-oligomerizes for efficient genome packaging, was greatly impacted by mutations throughout the pandemic, followed by changes in structure and intrinsic disorder. Remarkably, VOC constellations acted co-operatively to balance the more extreme effects of individual haplotypes. Our strategy of mapping the dynamic evolutionary landscape of genetically linked mutations to the N-protein structure demonstrates the utility of ab initio modeling and deep learning tools for therapeutic intervention.

## 1. Introduction

The Coronavirus Disease 19 (COVID-19) pandemic has led to a total of 775,645,882 reported cases and 7,051,876 deaths worldwide [1]. To put these numbers into perspective, the death toll alone is more than the populations of several US states such as Montana, Rhode Island, Delaware, South Dakota, North Dakota, Alaska, the District of Columbia, Vermont, and Wyoming combined [2]. The total number of cases exceeds the population of the entirety of Europe by ~28 million people [3]. This makes COVID-19 one of the deadliest pandemics to hit the human population in the 21st century surpassing the impact of the Middle East Respiratory Syndrome (MERS) (September 2012, Jeddah, Saudi Arabia) and Severe Acute Respiratory Syndrome (SARS) (March 2003, Guangdong, China) coronaviruses [4,5], which had a total death toll of 774 and 949 deaths worldwide, respectively [6,7]. This is remarkable because COVID-19 has a much lower mortality rate of 5.19% compared to 13% for SARS and up to 35% for MERS [8]. The disproportionate death tolls amongst these pandemics caused by coronaviruses can be attributed to the asymptomatic phase of COVID-19, which SARS and MERS lack [9]. Asymptomatic patients can carry on with their daily routines without even knowing that they are carriers of similar viral loads as their symptomatic counterparts, enabling high transmission rates within a population [10]. This challenges disease control and mitigation as asymptomatic individuals spread disease to the uninfected. The most extreme case of asymptomatic COVID-19 occurrence was the “Diamond Princess” cruise ship population in Japan, which experienced 641 COVID-19 infections out of 3711 passengers, with over half (328) of those infected being asymptomatic [11]. This makes the personal responsibility of an individual the ‘number-one’ factor in preventing the spread of COVID-19 through masking and other precautionary measures [12] and poses an even greater challenge for policymakers to ensure the proper measures are in place to prevent the spread of COVID-19.

The COVID-19 disease is caused by the Severe Acute Respiratory Syndrome Coronavirus 2 (SARS-CoV-2). The virus has a 79.6% sequence similarity to SARS-CoV-1, the causative agent of the first SARS outbreak [13]. Remarkably, 96% of its full-length ~30 kb genome sequence is identical to the sequence of a bat coronavirus [13]. The SARS-CoV-2 genome encodes 29 proteins, including 4 structural proteins crucial to viral function, the spike (S), nucleocapsid (N), membrane (M), and envelope (E) proteins. The evolution of the S-protein and M-protein and its effects on their structure have been previously reported [14,15]. In contrast, no significant changes were detected in the structure of the E-protein. The N-protein packages the positive-sense RNA genome of the virus forming the ribonucleoprotein structures of the viral capsid. The protein is necessary for viral assembly and RNA synthesis and participates in several cellular processes affecting immunological and cell cycle responses of the host [16]. The first mutations targeting the N-protein were found to be genetically linked via a haplotype (haplotype H2) [17]. The worldwide appearance of H2 very early in the pandemic followed the spread of the first reported haplotype (H5), which harbored the S-protein ‘D614G’ mutation. The N-protein comprises five distinct regions, two of which are structured and are known as the N-Terminal Domain (NTD) (residues 44–174) and the C-Terminal Domain (CTD) (residues 255–364). These domains are connected together by a disordered linker region (LKR) (residues 175–254) and are flanked by Intrinsically Disordered Regions (IDRs), the N-arm (residues 1–43), and the C-tail (residues 365–419) [18]. The CTD allows the N-protein to self-associate to form dimers while the C-tail mediates higher-order assembly into tetramers [19]. The disordered nature of the IDRs plays a crucial role in binding to viral RNA with high co-operativity by enhancing binding affinity and allostery [20]. This increased binding affinity to nucleic acids is the result of the flexibility of disordered regions, which allow for multiple nucleic acid-binding sites to associate with the same nucleic acid in an optimized allosteric conformation [21]. Both the NTD and CTD bind to viral RNA to form the nucleoprotein core of the virus [18,22]. The N-protein also interacts with the M-protein to fix ribonucleoproteins to the viral membrane [23]. Aside from its role in viral genome packaging, the N-protein also regulates antiviral immunity by the induction of interferon responses [24]. Following the formation of the ribonucleoprotein core at the ER-Golgi intermediate compartment (ERGIC) where the rest of the structural proteins are assembled, the budding process begins with proteins and viral RNA entering the lumen of secretory vesicles followed by transport of assembled virions out of the host cell via exocytosis [25,26,27]. There is considerable co-operativity between the four structural proteins of SARS-CoV-2. This enables efficient viral development and release, which is also demonstrated by the mutational evolutionary landscape. Many of the mutations observed have been dominated by the S, M, and N proteins throughout the pandemic [14]. These proteins are therefore important targets for vaccine and drug development.

Variants of Concern (VOCs) have been replacing each other since the beginning of the COVID-19 pandemic [28]. Their mutant constellations hold ‘mutations of concern’ that are of immediate priority for surveillance and response. The effect of these mutations on the protein sequence must be linked to effects at the three-dimensional (3D) atomic structure level to dissect the functional significance of individual VOCs and associated haplotypes. Three main strategies model protein structure: homology modeling, fold recognition, and ab initio methodologies [29]. Homology modeling and fold recognition rely on existing sequence and folded structure data and are rather comparative in nature. These methods can be limited in their ability to accurately predict the true 3D atomic structure of novel proteins, especially in molecular systems subjected to fast mutation rates. Ab initio methods, however, do not use pre-existing knowledge. Instead, they build models directly from amino acid sequences and the stoichiometric constraints of those sequences. Such an approach is especially useful for modeling proteins with low homology. These methods explore the 3D conformational space of a particular protein sequence with a set of rules [30]. An exploratory algorithm uses protein backbone and side-chain information to simulate a time series of protein-folding events [30]. The method involves a scoring function, which must differentiate native structures from non-native ones and an efficient method of searching the protein conformational space [30]. Initially, ab initio methods were very limited and computationally expensive but saw gradual improvements each year. AlphaFold2 [31], the star of the last two biannual ab initio structure prediction experiments (CASP 14 and 15) [32,33], greatly broke records as its neural network (called the *evoformer*) approach to modeling proteins without homologs was within the margin of error of experimental structure determination methods [31]. AlphaFold2 first builds the search space by creating a multiple sequence alignment (MSA) and pair representations of the input amino acid sequences, which are then processed by the *evoformer* to build models of the 3D structure. An iterative process known as recycling (three iterations by default or as set by the user) gradually refines the MSA, pair representations, and 3D structures until no further improvements can be made. The AlphaFold2 neural network was trained on the data available in the PDB database up until 30 April 2018, and five different models with random seeds search the 3D conformational space for model diversity, each with its own confidence score [31].

AlphaFold2 crucially reduces reliance on traditional crystallographic and cryo-EM methods that are time-consuming. In the absence of experimental protein structures from VOCs, numerous studies utilized AlphaFold2 to explore the differences between the wildtype (WT) Wuhan strain, which is used as reference, and the emerging variants [15,34,35,36]. However, variant definitions do not reflect the complete viral landscape of SARS-CoV-2. Other groups of mutations can occur in greater frequencies than VOC constellations, and VOCs often embody latitude-delimited haplotypes that have their own unique accumulation profiles across climatic zones [14,37]. These haplotypes, which were identified following a study of over 12 million viral proteomes, uncover seasonal patterns of emergence and help link structural conformations to environmental factors revealing an interplay between viral evolution, our environment, and our immune systems [14]. In fact, COVID-19 epidemiological variables exhibit significant negative correlation with temperature and positive correlation with latitude, often peaking around winter months, further indicating the seasonal nature of the virus [37,38,39].

Despite advances, a comprehensive study of structural changes induced by haplotypes on the N-protein is absent from the current literature with most research being focused on the VOCs [40,41]. These studies also shed light on the utility of AlphaFold2 to explore how the N-protein would fold in its entirety, as to date, no experimental structures exist for the complete protein (only the NTD and CTD domains have been determined) [40]. Studies also at times include a structural comparison considering individual mutations or groups of mutations passing a certain threshold after processing sequences from GISAID (https://www.gisaid.org/, accessed on 1 July 2024) [40,41,42]. This still does not encompass the coupling and decoupling of mutations within haplotypes and variants that are occurring across the globe due to evolutionary pressures such as climate or global vaccination campaigns. Here, we use AlphaFold2 to model the 3D structures of mutant N-protein molecules defining SARS-CoV-2 haplotypes and constellations (Table S1), filling the need of a comprehensive exploration of their impact on protein structure. We study the effect of mutations on the regions of intrinsic disorder of the molecule, studying structural, flexibility, and electrostatic changes across the pandemic. Our study uncovers patterns of structural recruitment across haplotypes and VOCs indicative of a complex interplay between the virus and its environment that mediates viral evolution. We show the N-protein was impacted by many haplotypes, beginning with H2 and its effects on the LKR region and ending with the rise of the VOC Omicron constellation and co-operative effects on protein structure. Our study highlights the importance of ab initio techniques and the utility of AlphaFold2 for comparative structural studies of proteins without any experimentally determined atomic structures and containing regions of intrinsic disorder known to be difficult to model by traditional means.

## 2. Materials and Methods

Accelerated ab initio modeling of 3D atomic structures of the N-protein was conducted using the AlphaFold2 pipeline [31] implemented locally in ColabFold without changes or modifications [43]. The output of five ranked structural models was obtained following twelve neural network recycles (processing of predictions through models) that iteratively extracted co-evolutionary information in PDB70 structural templates and multiple sequence alignments (MSAs) for end-to-end training of the deep learning ‘evoformer’ and ‘structure’ multi-layered neural network modules. MSAs were built with fast and sensitive MMseqs2-based homology searches of UniRef100 and a database of environmental sequences. Accuracy was measured with the predicted local-distance difference test (pLDDT) and the predicted aligned error (PAE). pLDDT provides a per-residue estimate of prediction confidence based on the LDDT-Cα metric [44]. The expected prediction reliability of a given region or molecule follows pLDDT ‘confidence bands’: >90, models with very high confidence; 90–70, models with confidence, showing good backbone predictions; 70–50, models with low confidence; and <50, models with very low confidence, generally showing ribbon-like structures. pLDDT < 60 can be considered a reasonably strong predictor of intrinsic disorder. PAE measures confidence in the relative positions of pairs of residues, which evaluates the cohesiveness of structural modules (e.g., domains).

Structural alignments and visualizations were carried out using Chimera [45]. Reference (corresponding to EPI_ISL_402124) and variant structures were superimposed using the MatchMaker and MatchAlign tools to identify regions with structural divergences. Topological similarities of individual regions or entire molecules were evaluated with average template modeling scores (TM scores) using US-align [46,47]. Electrostatic (Coulomb) potentials were traced on surface representations of the structural model using surface-coloring implementations in Chimera. Coloring involves calculating electrostatic potentials according to Coulomb’s law. Potentials depend on atomic partial charges and a distance-dependent dielectric constant and are calculated as sums of potentials for atoms in molecular surfaces. Electrostatic surface coloring using more complex Poisson–Boltzmann calculations provides remarkably similar tracings and was not implemented. To quantify protein flexibility and other dynamic features across the pandemic, the Bio3D [48] package in R [49] was used to obtain fluctuation values which were then visualized in python. Matplotlib [50], numpy [51], and pandas [52] in python were used to process and visualize the data retrieved from the tools used in this study. N-protein predictions were benchmarked against cryo-EM models of the two structural domains [18]. Besides TM scores, Global Distance Test-Total Score (GDT-TS) scores were obtained using the LGA (local–global alignment) structure comparative analysis tool with the AS2TS server [53,54,55], which CASP assessors routinely use to evaluate the accuracy of predicted structural models. The data presented in this study for the N-protein are openly available in ModelArchive under accession ma-gca-nprot (https://www.modelarchive.org/doi/10.5452/ma-gca-nprot; released on 16 July 2024).

## 3. Results

### 3.1. Full-Length Analysis of the N-Protein Using Backbone Root Mean Square Deviations (RMSD)

**Figure 1:**
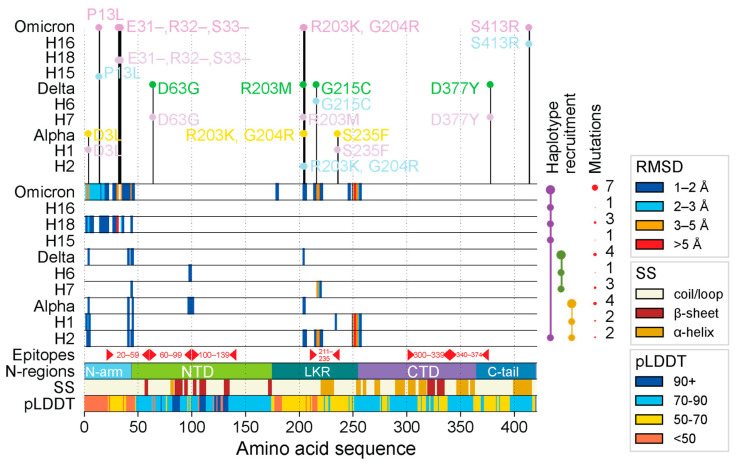
Regions of structural deviation. The vertical axis represents each of the haplotypes and variants, impacting the N-protein, arranged in chronological order from earliest to latest, up until the rise of VOC Omicron. The horizontal axis represents the amino acid positions along the length of each of the N-protein molecules. The horizontal bars representing the haplotypes and VOC constellations are colored in hues of light blue and thistle in an alternating fashion whereas VOCs Alpha, Delta, and Omicron are colored yellow, green, and purple, respectively. When a residue along the length of any of these protein molecules crosses the 1 Å, 2 Å, 3 Å, and 5 Å RMSD, then that position is colored in dark blue, light blue, orange, and red, respectively. The area labeled ‘epitopes’ includes residue ranges for important epitopes found along the length of the N-protein as reported by Smith et al. [58]. The horizontal bar labeled ‘N-regions’ indicates all positions of important regions/domains along the N-protein: N-arm (1–43), NTD (44–174), LKR (175–254), CTD (255–364), and C-tail (365–419). The horizontal bar labeled SS indicates the position of alpha helices (brown), beta sheets (dark red), and coils (beige). The horizontal bar labeled pLDDT represents the confidence level of AlphaFold2 for each residue along the S-protein. On top of the graph are the individual mutations that comprise each haplotype and variant.

The N-protein was significantly impacted by four haplotypes (H1, H2, H7, and H18) and VOC Omicron. Mutations affected the structure of four small regions of the molecule. Figure 1 visualizes the amount of structural deviation for each VOC and its corresponding haplotypes with respect to each residue in the N-protein structure. Individual structural alignments from Chimera using the MatchMaker tool were used to extract RMSD headers, which contained the structural deviation per residue in Ångstroms (Å) for the protein backbones. This allowed us to ascertain which areas along the length of the N-protein experienced the most change. Using an RMSD threshold of 3 Å (a standard for protein comparison and homologous proteins [56,57]), we observed that the N-protein exhibited little change for each haplotype and VOC, aside from affecting the four target regions. The most notable change occurred in the LKR region, which is also the region most heavily impacted by amino acid mutations. Haplotypes H2, H1, H7, and VOC Omicron affected the LKR structure at two specific locations (residues 210–220 and 247–257). Table S1 summarizes the mutations per haplotype/VOC along with their time of origin.

**Figure 2:**
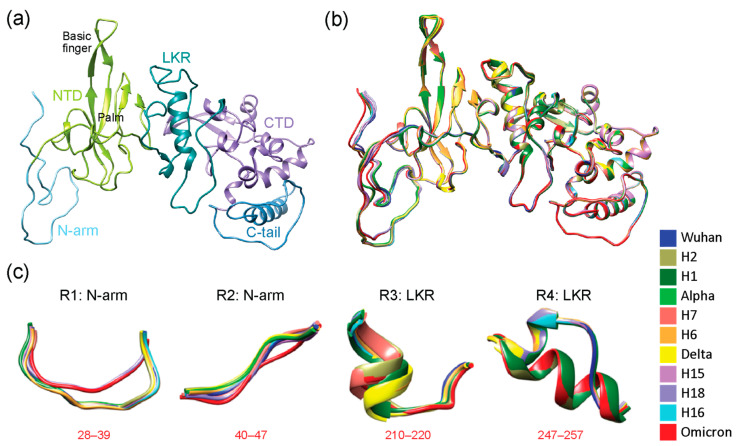
Three-dimensional (3D) models of molecular change at the atomic level in regions of structural deviation. (**a**) The N-protein modeled by local ColabFold for the Wuhan strain with its 5 structural domains color-coded as N-arm (light blue), NTD (lime green), LKR (teal), CTD (purple), and C-tail (steel blue), the same color scheme as the N-regions bar in Figure 1. Areas that saw structural deviation crossing the 3 Å threshold for each haplotype and VOC have been highlighted in red. (**b**) All haplotypes and VOCs were superimposed onto the Wuhan structure using Chimera and are color-coded according to the bottom-right index. (**c**) Using data from Figure 1, all residues that surpassed the 3 Å threshold and other regions from further inspection in Chimera were translated into 4 regions of structural deviation (R1 to R4). Model snapshots of these regions were taken in Chimera to ensure all of the superimposed structures, and their corresponding residues were adequately captured for each region. The location of regions in the amino acid sequence is indicated in red. Each variant and haplotype structure are color-coded according to the index.

Our results revealed that VOCs Alpha and Delta suppressed the individual effects of their constituent haplotypes, whereas VOC Omicron amplified these effects. One notable change occurred in the N-arm and was restricted to H18 and VOC Omicron due to the triple-deletion mutation (E31-, R32-, S33-), which significantly changed the structure of the region. In contrast, other haplotypes and VOCs showed more or less the same structure aside from slight shifts in 3D space. VOCs Alpha and Delta behaved antagonistically to the extreme effects of their haplotypes, while VOC Omicron behaved synergistically. However, each VOC tended to inherit non-significant structural changes, though these regions provided little added functional benefit due to their similarity. To further analyze these regions of structural deviation, snapshots of the structural alignment were taken in Chimera, and a total of four regions of interest (labeled R1–R4) were identified (visualized in Figure 2b,c). The first two regions were part of the N-arm and were adjacent to one another. Only H18 and VOC Omicron impacted the N-arm; we observed a shorter loop turn due to the triple deletion in R1 and a slightly tighter conformation of R2 when compared to other haplotypes and VOCs. R3 and R4 were located in the LKR. R3 showed a short alpha helix with the structures of H2, H7, and VOC Omicron being off-axis compared to the rest of the structures. R4 also involved an alpha helix with H1, H2, and VOC Omicron, adopting a much longer helix than the rest of the structures and causing them to have a very noticeable shift from the original Wuhan backbone.

The majority of the N-protein structure was disordered in nature. As shown in Figure 2, the N-arm (light blue), LKR (teal), and C-tail (steel blue) were largely composed of loops and coils. Conversely, the NTD (lime green) and CTD (medium purple) were structured. Aside from the D63G mutation, no other amino acid mutations occurred in the NTD or CTD regions, helping explain their structural stability. In contrast, the IDRs of the N-protein were the main hotspots for mutational and structural change. Of the three IDRs, the LKR saw the most mutations, with six positions being impacted compared to three in the NTD (triple deletion counted as one) and two in the C-tail. There were few changes in the beta-hairpin of the NTD, which fell under the 3 Å threshold. Figure S1 describes these regions in greater detail, with an atomic-level view of the differences between corresponding atoms of N-proteins from haplotypes and VOCs.

### 3.2. Regional Analysis with TM Scores Using US-Align

**Figure 3:**
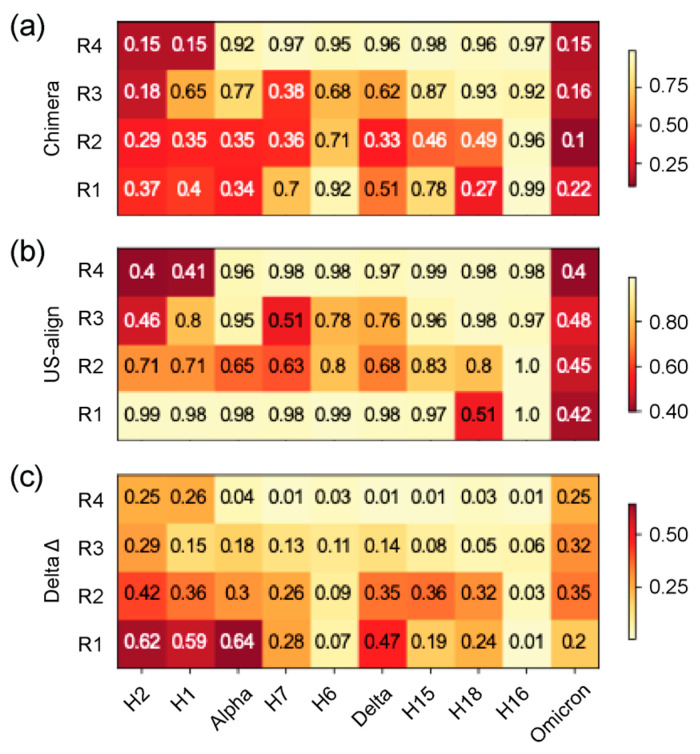
Heatmap of TM scores (ranging 0–1) of regions of structural domains. The 4 regions of structural deviation (R1–R4) were used to slice the corresponding regions from each file and obtain TM scores for each VOC and haplotype with US-align, using the Wuhan reference molecule as the template. The horizontal axis is arranged in chronological order depicting changes across the timeline of the pandemic, and the vertical axis represents the 5 structural domains described in Figure 3. All TM scores falling under the TM threshold of 0.5 are colored in light cream. (**a**) TM scores using alignments from Chimera superimposition. (**b**) TM scores using US-align alignments. (**c**) Difference between heatmaps in (**a**,**b**).

TM scores from the Universal Structural Alignment (US-align) program [47] were used to better analyze the structural homology of the four regions of structural deviation (R1–R4) of the N-protein for each haplotype and VOC against the Wuhan structure. This similarity measure is superior to recording single-residue RMSD changes, which are very sensitive to small changes in conformation. The four regions from each haplotype and VOC were sliced out from their original ‘pdb’ files and compared to the Wuhan regions separately. Figure 3 reports these results as a heatmap with their corresponding TM score in each cell. Darker colors indicate higher structural deviation than lighter colors. Two alignments were used for this analysis, one from Chimera’s superimposition (Figure 3a) and a second further alignment forced by US-align (Figure 3b). This allowed us to judge if a structural change was more than just a shift in 3D space impacting instead the morphology of the region altogether.

In the heatmap, we revealed numerous values under 0.5, the minimum score required for proteins to be considered in the same fold in the Structural Classification Of Proteins (SCOP) and Class, Architecture, Topology and Homologous superfamily (CATH) databases [46,59]. There were significant changes observed for the four regions throughout the pandemic. However, when forcing a second alignment in US-align, many values shifted to above 0.7, indicating that those changes were only shifts in 3D space and were most prevalent for R1, which only saw H18 and VOC Omicron retain TM scores below 0.5. In contrast, more than half of the structures had scores lower than or close to 0.5. R2 was similar to R1, but this time, only VOC Omicron retained a value close to 0.5 but had generally lower TM scores than R1. R3 showed very consistent results throughout both alignments, indicating changes in these regions were more morphologically relevant, with H2, H7, and VOC Omicron sharing similar conformations. R4 stood out for its consistency as well, with almost every other structure having a TM score over 0.9 and only H1, H2, and VOC Omicron revealing scores under 0.5 that indicate the targeted nature of this structural inheritance across the length of the pandemic.

### 3.3. Protein Disorder and pLDDT Scores

**Figure 4:**
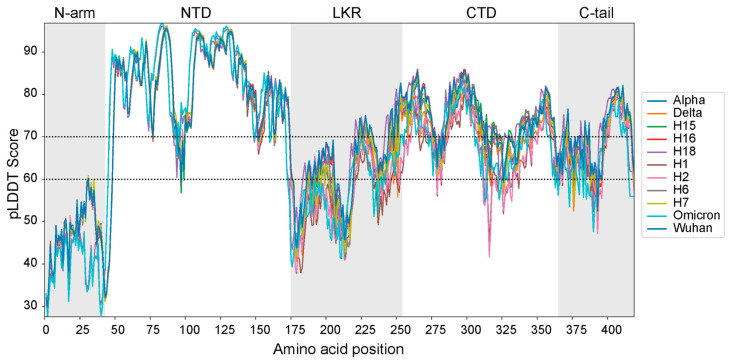
Per-residue pLDDT scores of each AlphaFold2 top-ranked modeled 3D structure.

Regions R3 and R4 were remarkable because they attained secondary structures in primarily disordered segments. Using the confidence metric of AlphaFold2, the pLDDT score, we showed that these regions had lower values (Figure 4). This is expected. IDRs are known to exhibit lower pLDDT values. In fact, AlphaFold2 now uses pLDDT scores to predict regions of disorder [60]. The lower pLDDT scores of R3 and R4 likely indicate the transient nature of helices, where they exist only briefly and could be part of a short linear motif [61,62]. The three IDRs in the N-protein had notably lower pLDDT scores than the ordered NTD and CTD regions. The NTD was the most structured region with most of its residues scoring around 90 or 80. Only residues 94 to 98 scored below 70. The CTD was the second most ordered region. It showed lower scores than the NTD but still above 70 for most of its residues. Exceptions included the region spanning residues 312 and 336 and the residues close to the C-tail. Almost the entirety of the residues of the N-arm showed scores below 60. Surprisingly, a small portion of the LKR structure showed scores above the 70 pLDDT threshold in regions spanning residues 223, 232, 244 and residues at the end of the region. The C-tail had a significant portion above 70, starting from residue 383, which runs towards the end of the structure, with a few smaller regions also scoring 70 towards the start of the C-tail. The N-arm behaved exactly as one would expect from an IDR. However, the LKR and C-tail exhibited some regions of high confidence. Note that IDRs that have high pLDDT scores from AlphaFold2 can undergo conditional folding under certain conditions to interact with other proteins [63]. Thus, the LKR and C-tail regions are potentially involved in molecular interactions.

### 3.4. Protein Disorder and Binding Capacity Across the Pandemic

**Figure 5:**
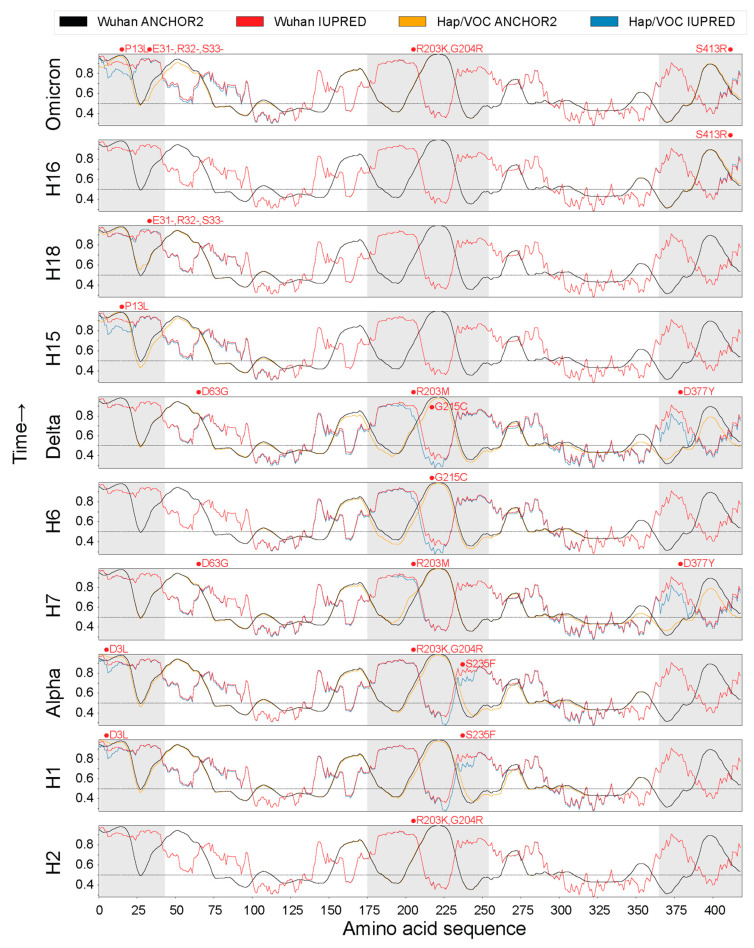
Intrinsic disorder and binding capability across the pandemic.

Protein disorder can play a crucial role in viral function. Therefore, we explored if disorder had changed or evolved during the pandemic. Using the online tool IUPred2A, a combination of Intrinsically Unstructured protein Prediction (IUPred) and ANCHOR [64], we measured the impact of the haplotypes and VOCs on intrinsic disorder and binding capability across the length of the N-protein. In Figure 5, the IUPred scores (a measure of protein disorder) and Anchor2 scores (a measure of binding capability) are plotted for each haplotype and VOC in chronological order from Haplotype H2 (bottom) to VOC Omicron (top). Despite their mutations, H2, H18, and H16 exhibited almost no or negligible change for both the IUPred and Anchor2 scores.

Overall, we saw decreases in protein disorder across the pandemic, along with decreases in binding capability for the other haplotypes and the three major VOCs. There were increases in binding capability for H1 and VOC Alpha around residue position 235 due to mutation S235F and for H7 around residue position 203 due to the mutation R203M. Significant changes to protein disorder and binding capability were also limited to the three IDRs, with most of them occurring in the LKR during the H1 to VOC Delta phases of the pandemic. Conversely, the latter half of the pandemic saw most of its changes at the N-arm. VOC Delta and its corresponding haplotypes characteristically impacted the LKR and C-tail, whereas VOC Alpha and its haplotypes targeted the N-arm and LKR. The most notable differences included the N-arm (H1, VOC Alpha, H15, VOC Omicron), LKR (H1, VOC Alpha, H6, VOC Delta), and the C-tail (H7 and VOC Delta). This pattern of changing levels of protein disorder in specific regions of the N-protein indicates the changing needs for viral survival. As with the antagonistic and synergistic recruitment patterns of the various substructures along the protein and across the pandemic, we reveal a similar effect with protein disorder and binding capability in these studies. The interplay between the structural conformations and biochemical properties of these regions can help us understand how these different protein structures are adapted to interact with other proteins for effective viral functioning. This can be achieved through, for example, forthcoming Molecular Docking simulations.

### 3.5. Normal Mode Analysis of Haplotypes and VOCs

**Figure 6:**
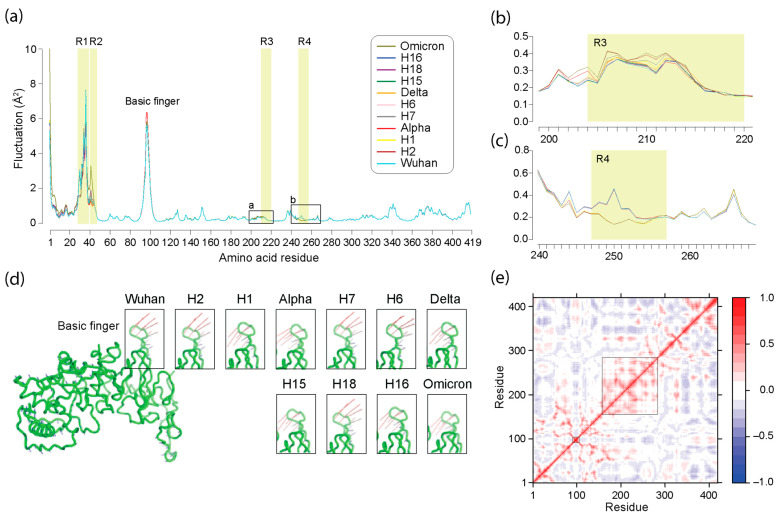
Normal mode analysis (NMA) of the N-protein across the pandemic. (**a**) Atomic fluctuation of residues mapped along the amino acid sequence of the N-protein for haplotypes and VOCs. Regions of structural change (R1, R2, R3, and R4) are shaded in light yellow, and the location of the nucleic acid-binding ‘basic finger’ is identified. (**b**,**c**) Details of plots in regions surrounding the R3 and R4 regions. (**d**) Visualization of the first non-trivial mode (mode 7) of the Wuhan N-protein molecule reflecting optimal energy states using a vector field representation. The most salient motion pattern in the ‘basic finger’ is highlighted and unfolded for all haplotypes and VOCs, showing notable changes in motion directionality. (**e**) A dynamic cross-correlation heat map for the Wuhan molecule averaged over all modes show correlated (red hues) and anti-correlated (blue hues) regions in the protein structure. Big and small boxes highlight the positively correlated LKR sequence embedding the R1 and R2 regions and the ‘basic finger’, respectively.

Normal mode analysis (NMA) is a computational technique used to study protein dynamics in disordered systems and correlate movements along a protein molecule [65]. Normal modes reflect patterns of molecular ‘vibration’ that manifest as oscillating conformational changes. NMA is very computationally inexpensive and well suited for smaller molecules [66]. However, its application has not been widespread. Here we used NMA to quantify the flexible states available to the N-protein for each haplotype and VOC using the fluctuations metrics from Bio3D [67]. These metrics are based on atomic fluctuations of the amino acids as these are being impacted by side-chains, proximity to other parts of the molecule, and more [68]. High distance fluctuation values indicate higher flexibility, whereas lower fluctuation values indicate lower flexibility. Figure 6 summarizes the important differences observed for each haplotype and VOC. With an exception in N-terminal residues 1–3 and 86–98 of the N-protein, the majority of changes in accessible conformational states (flexibility changes measured as atomic distance ‘fluctuations’ in Å^2^) of significance in haplotypes and VOCs were observed occurring in regions of structural deviation R1 (residues 28–39), R2 (residues 40–47), R3 (residues 210–220), and R4 (residues 247–257). Thus, structural changes in haplotypes and VOCs result in changes in accessible conformational states defining molecular motions, with R1 and R2 changes in the N-arm being directly associated with them. Flexibility changes in the first three residues of the molecule showed that VOC Omicron had noticeably higher fluctuation values starting at 10 Å^2^, with all the other structures not exceeding 6 Å^2^. Fluctuations associated with residues 86–98 formed a peak that reached 6.5 Å^2^ levels and were maximal in VOCs Alpha and Delta. This area of the molecule ends in a loop that subtends a crucial RNA-binding protuberance located in the middle of the NTD (also known as the ‘basic finger’) [22,69]. Fluctuation values in R1 and R2 ranged from 2 to 7 Å^2^. VOC Omicron and H18 showed higher fluctuations, but with H18 being lower than the others around residues 34 and 35. Fluctuations in residues 40 to 44 of R2 showed only slight deviations from the Wuhan reference except for VOC Omicron. Fluctuation values past residue 100 were nominal (never exceeding 1) and typical of many protein molecules (e.g., lysozyme). However, changes in structure resulted in notable changes in atomic fluctuation (Figure 6b,c). Fluctuation for VOC Omicron and H18 were higher than the Wuhan baseline for residues 200 to 205, followed by VOC Alpha. Fluctuations for VOC Omicron and H18 again remained higher than the others for residues 206 to 216, which embed R3, followed by H7 which intermittently reached either higher values or the Wuhan baseline. Conversely, fluctuations for H18 and H16 remained higher than for others in residues 243 to 255 that span R4.

The first non-trivial mode or the lowest normal mode is a useful indicator of protein flexibility as it is able to capture information about the most mobile parts of a protein along with movement direction [70]. This normal mode (mode 7) of the N-protein structures, which depicts molecular bending, was visualized with vector field representations, in which vectors for each amino acid residue specified the magnitude of its molecular vibration (a molecular ‘trajectory’). Figure 6d, which summarizes mode 7 vector field representations for all modeled structures, reveals that the most significant motion that was detected was associated with the ‘basic finger’ of the molecule (see also Figure S2). The ‘basic finger’ exhibited changes to the directionality of motion throughout the pandemic. All VOCs along with haplotypes H1, H15, and H16 had ‘basic fingers’ moving in the opposite direction to that of the Wuhan structure. For VOCs Alpha and Omicron, it can be said that their haplotypes contributed to motion inversion. In contrast, haplotypes H7 and H6 of VOC Delta appear to provide a hidden cumulative effect that later materialized in the structure of their VOC. This coupling–decoupling of motions describes the impact of regions of structural change on the dynamic landscape of molecular motions.

Dynamic cross-correlation patterns identify motions that are correlated to those in other parts of the molecule. Figure 6e shows a cross-correlation map for the Wuhan N-protein averaged over all modes. The map is almost indistinguishable from maps generated for haplotypes and VOCs (Figure S2), illustrating the well-known fact that distinct correlated movements identified when analyzing individual modes can cancel each other when averaged over all modes. The cross-correlation matrix shows that areas embedded in the intrinsically disordered LKR, which hold R3 and R4, and the ‘basic finger’ are generally strongly correlated with each other but often negatively correlated with the rest of the molecule. This property defines a cohesive dynamic behavior governing their motions, which appears puzzling for a disordered region.

### 3.6. Exploring Electrostatic Potential Surface Fingerprints

**Figure 7:**
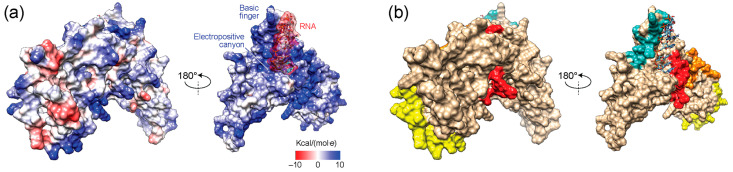
Electrostatic surface potentials of the modeled Wuhan reference N-protein bound to a 7 mer RNA molecule (5′-CUAAACG-3′). (**a**) The electrostatic (Coulomb) potential surface representation of two sides of the molecule reveals a largely electropositive molecular face with basic region largely matching the colored RNA-binding regions described in the other panels. (**b**) Surface representation with known RNA-binding structural elements colored in teal (basic finger of the NTD), red (SR-region of the LKR), orange (N-terminal region of the CTD embedding R3), and yellow (C-terminal region of the C-tail).

Electrostatic surface potentials provide insights into possible interactions of the molecular surfaces of the N-proteins of variants and haplotypes. One main role of the nucleocapsid molecules is to bind to the single-stranded RNA viral genome, which has potentials that are chiefly electronegative. These potentials counteract electropositive regions of the nucleic acid substrate. Figure 7 shows an electrostatic (Coulomb) potential surface representation of the reference Wuhan N-protein that we modeled. The molecule was aligned to the lowest energy NMR structure of a SARS-CoV-2 NTD in complex with a 7 mer single-stranded RNA [69], which was deposited under PDB accession 7ACS. The experimentally acquired NTD structure, which aligned well to the modeled N-protein structure (RMSD = 1.04 Å; 103 pruned atom pairs), was then deleted for visualization of molecular surfaces interacting with the nucleic acid. The N-protein had two faces. One had surface potentials that were mostly neutral but with three major areas that were fundamentally electronegative located in the C-tail and numerous small areas that were electropositive and spread throughout the face of the molecule. The other face had surface potentials that were largely electropositive and served as large nucleic acid-binding areas. In fact, known RNA-binding elements [22] traced on the surface of the molecule spanning the ‘basic finger’ of the NTD domain and the C-tail of the molecule (Figure 7b) matched the most salient electropositive regions of the molecular face (Figure 7a). In particular, the binding interface between the NTD and the backbone of the single-stranded RNA molecule was localized in a electropositive ‘canyon’ between the ‘basic finger’ and the ‘palm’ helical sheet of the NTD, which embeds three arginine residues (R92, R93, and R149) and a lysine (K102) known to bind to RNA [69].

**Figure 8:**
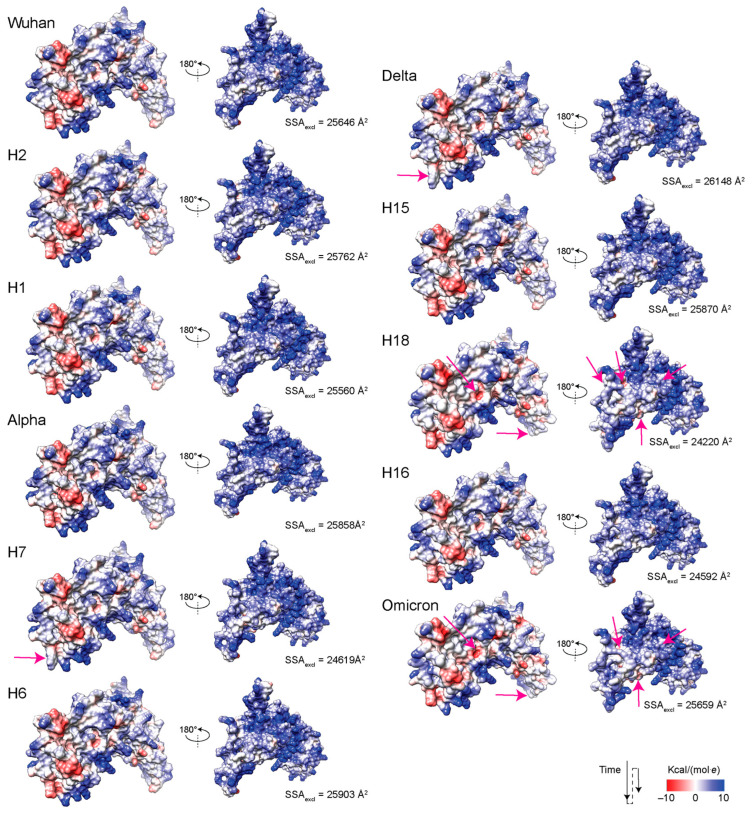
Electrostatic surface potentials of the N-protein from haplotypes and VOCs. Areas exhibiting electrostatic change are indicated with pink arrows. SSA_excl_, solvent excluded surface area.

Figure 8 shows surface representations of electrostatic potentials for the molecules of haplotypes and VOCs. Overall, very few significant changes altered the electrostatic surface of the molecules, the first appearing in H7 and then VOC Delta as a loss of an electronegative region in the C-tail of the non-binding face. These changes disappeared with the rise of new haplotypes and VOCs. Six significant changes were observed in H18 and VOC Omicron, one affecting N-tail and four affecting the binding face of the molecule. One of these changes altered the nucleic acid-binding SR-region of the LKR, which is downstream of R3 and close to R4 (Figure 7).

### 3.7. Benchmarking Alphafold2 Reference Structures against Experimental Cryo-EM Models

To validate our findings, we compared the AlphaFold2 structures against experimental crystallographic atomic models of the N-protein [18]. These structures were limited to the NTD and the CTD regions as the other regions of the N-protein were very disordered [71] and difficult to model. Since the N-protein has the ability to self-associate and form tetramers mediated by the CTD [19], we benchmarked the AlphaFold2 Wuhan model (with the NTD and CTD regions sliced out for independent comparisons) against the four chains of the NTD and the CTD provided by Peng et al. (2020) [18]. These models are available in the PDB database [72,73] with the following IDs: 7CDZ for the NTD (doi.org/10.2210/pdb7cdz/pdb) and 7CE0 for the CTD (doi.org/10.2210/pdb7ce0/pdb), each with four chains labeled A to D [18]. Amino acid sequence to tertiary structure (AS2S) [54] and US-align [47] were used to assess the quality of the AlphaFold2 model against the experimentally determined structures, and the results were reported in Table 1. The NTD shows very good structural homology with the experimental structures, obtaining GDT_TS scores around ~90 which can be thought of as the percentage of the protein that was modeled correctly [53], and the TM scores are also very high, being above 0.9 for each chain. The AlphaFold2 model of the NTD also remained under an RMSD of 1.5 Å for more than 96% of its structure (126/131 residues) as reported by the AS2S server. These scores indicate very good structural topologies with very confident side-chain conformations that can be used for further research and analysis. They also justify the haplotypic analysis of the NTD region. The CTD region, however, had lower GDT_TS and TM scores than the NTD but remained slightly below 70 and ~0.74, respectively, which are still adequate for use in research as both these scores indicate accurate topologies but less confidence with side-chain conformations.

## 4. Discussion

The N-protein is responsible for binding to the genetic material of the virus (RNA) and packaging it into ribonucleoprotein (RNP) particles [74]. This is achieved through RNA-binding at various locations of the N-protein, which spans the N-arm [75], NTD [69], LKR [75], and CTD [20,76,77] regions. The CTD is also responsible for tetramer formation due to its ability to self-associate with the aid of the C-tail region [19]. The LKR plays an important role in RNP packaging [78] and oligomerization of N-protein molecules [79]. Both of these functions are achieved through biochemical and physical changes brought about by the phosphorylation of the region, which introduces significant electrostatic changes that are required to mediate these processes [74]. We observed that the most significant structural changes induced by haplotypes and mutant constellations occurred in the LKR region across numerous phases of the COVID-19 pandemic. In contrast, the NTD only saw significant change (including morphological changes apart from shifts in 3D space) in H18 and VOC Omicron due to the triple deletion. Note that both regions R1 and R2 of the N-arm are part of a single epitope ranging from residues 20–59, and R3 is part of an epitope ranging from residues 211 to 235 [58]. R1 mostly sees significant structural changes in H18 and Omicron as both of those structures harbor the triple deletion which all other structures lack (Figure S1). This suggests that the structural and mutational changes we observed could be due to the immunological pressures acting throughout the pandemic, forcing the virus to evolve to better ensure its survival. R3 is especially important because not only do we observe structural changes, but they are accompanied by the existence of an epitope that also saw two mutations in the region, including G215C and S235F, exhibiting notable decreases in protein disorder (Figure 5). Our findings suggest that structural changes are observed in regions of functional significance that help immune evasion and other viral functions, including oligomerization for effective genome packaging.

The N-protein is already a very disordered protein, and hence, changes are more likely to occur in regions of disorder. However, it is apparent that the NTD- and CTD-structured regions remain relatively unchanged in sequence and structure except for one mutation (D63G) in the NTD (Figure 1). Interestingly, the most common trend observed was that both protein disorder and binding capability actually reduced for most regions of the N-protein throughout the pandemic with the only notable increase being observed in the LKR for H1, H7, and VOC Alpha. The increase in binding capability was inherited from H1 to Alpha. However, the same was not true for VOC Delta and H7. Haplotypes H7 and H8 seemed to cancel out the impact of the R203M mutation on binding affinity, and both R203M and G215C removed the increase in binding capability at the LKR (Figure 5). This portrays the complex interactions between different mutations of several haplotypes within their VOC constellations as they can behave synergistically or antagonistically toward one another. Despite these changes occurring in the regions of disorder, all of the changes we reported showed a decrease in protein disorder except for one very small portion at the triple-deletion site in H18 and VOC Omicron.

R2 and R3, which embody established epitopes [58], were the only two regions that saw structural changes that were of immunological significance. Both of these regions showed decreases in protein disorder accompanied by decreases in binding capability (Figure 5). This indicates a mechanism of immune evasion where mutations such as the triple deletion in H18 and VOC Omicron, along with the mutations G215C and S235F, acted as a means to bind less effectively with antibodies. These structural changes, along with those in R1 and R4, appear to be recruited throughout the pandemic in various combinations amongst haplotypes and VOC Omicron. This combinatorial strategy highlights the evolutionary landscape of structural exploration that is unfolding in the N-protein, where combinations of smaller structural changes are adopted that benefit viral function. These changes are not limited to phases of the pandemic either. Earlier structures such as those observed in region R4 of haplotypes H1 and H2 were later found in VOC Omicron. In Figure S1, we observe that H1, H2, and Omicron have an alpha helix causing significant differences in atomic distances between corresponding atoms. Similarly, R3 revealed a similar recruitment pattern spanning the entire length of the pandemic, with the same substructure being found in H2, H7, and VOC Omicron. H2, H7, and VOC Omicron have an extended alpha helix that is part of the bigger alpha helix downstream of the structure and is slightly shifted from the original axis of the Wuhan structure (Figure S1). H1 and VOC Delta also adopted extended alpha helices in R3 but were not shifted from the original axis. The exploration of various substructures at specific regions of a protein can be thought of as a metric of structural entropy where higher numbers of structural conformations indicate higher levels of structural entropy. Most of the changes in the N-protein did not show much variation in the substructures but only in the combination of these substructures with one another. Since the same structure was recruited at regions R3 and R4 along the length of the pandemic, we consider these to be examples of entropic fixations. Here, structural entropy does not expand as numerous conformations are not being explored, indicating the N-protein adopted a more targeted evolutionary approach when compared to the S-protein [15]. These structural recruitments reveal patterns of synergy and antagonism during the pandemic. VOCs Alpha and Delta behaved antagonistically to the structural effects of their constituent haplotypes undoing their structural impacts. VOC Omicron, in turn, amplified or retained the individual effects of its constituent haplotypes. These effects mimic those found in our study of the S-protein [15].

Finally, an NMA of protein dynamics revealed that regions of structural change in haplotypes and VOCs were also coupled to patterns of molecular vibration indicative of molecular flexibility. These couplings also showed patterns of synergy and antagonism. Regions R1 and R2 exhibited significant molecular fluctuations, while those in R3 and R4 showed nominal values typical of many proteins. One notable molecular motion that was not associated with regions of structural change occurred in a protuberance known as the ‘basic finger’ of the NTD, an electropositive region involved in the binding of RNA that channels the molecule into an electropositive ‘canyon’ delimited by its ‘palm’ region [69]. Electrostatic potential surface fingerprints revealed several notable decreases in electropositive regions in H18 and VOC Omicron, which affected the nucleic acid-binding face of the molecule. One of these changes altered the nucleic acid-binding SR-region of the LKR, which locates between R3 and R4 (Figure 7). Vector field representations showed the direction of movement trajectories of the ‘basic finger’ in the reference Wuhan molecule changed in VOCs Alpha, Delta, and Omicron. Regions identified as very flexible and high energy from NMA tend to be more correlated with their surrounding amino acid residues and anti-correlated with residues farther apart from them. Additionally, the ‘basic finger’ and regions R3 and R4 which stood out from the NMA analysis also had great functional significance to the N-protein, which can indicate the utility of NMA in finding regions of functional importance to a protein. Results uncovered coupling–decoupling of motions indicative of long-distance effects of structural change on the dynamic behavior and electrostatic surface potential of the N-protein.

Our study covers numerous aspects of protein structural and functional analysis. Using protein disorder (from IUPred2A [64]) and low-energy modes (from NMA using Bio3D [67]) aid protein comparison studies in their search of regions of interest associated with protein bending, movement, and function. Exploring protein electrostatic surface potentials, which directly impact protein-folding and binding activities, complement these studies. 

## 5. Conclusions

One remarkable finding of our ab initio modeling exercises is that VOC constellations counteracted the more extreme effects of individual haplotypes on protein structure. This reveals a difference in evolutionary pressures for different proteins and showcases how haplotypes can be more or less beneficial for their corresponding VOCs. There also exists a pattern of recruitment where different haplotypes and VOCs adopt similar conformations at specific sites across the pandemic indicating the importance of both structural and mutational entropy [17]. The virus explores various structural changes during the evolutionary process to find the combination of structural conformations that best suit survival and fitness. This strongly suggests that a co-operative activity exists in haplotype-mediated protein communication, which was already made explicit in previously described haplotype-delimited protein interaction networks [14]. The impact of haplotypes H1 and H2 on the N-protein now reveals seasonal effects in mutation accumulation patterns along the length of the pandemic [14]. H1 achieved the highest prevalence in Arctic and Northern temperate regions indicating temperature sensitivity. H2 showed a seasonal pattern of accumulation, which later turned into complete prevalence during the pandemic. This information will be especially valuable for therapeutic interventions and predictive intelligence applications, which could facilitate understanding of protein reformation, de novo protein and construct design, and assembly of molecular complexes.

## Figures and Tables

**Table 1:** Benchmarking results.

State-Chain	GDT-TS (AS2S)	TM Score/L (US-Align Server)	Superimposed RMSD < 5 Å (AS2S)
NTD-A (7CDZ)	91.99 × (128/131) = 89.88	*L1: 0.93398, L2: 0.91375	1.293/128
NTD-B (7CDZ)	90.35 × (127/131) = 87.59	0.92756, 0.90091	1.366/127
NTD-C (7CDZ)	95.24 × (126/131) = 91.60	0.95983, 0.92447	0.941/126
NTD-D (7CDZ)	94.26 × (122/131) = 87.78	0.93300, 0.90592	1.1108/122
CTD-A (7CE0)	69.73 × (109/110) = 69.10	0.74156	2.508/109
CTD-B (7CE0)	69.50 × (109/110) = 68.87	0.73968	2.519/109
CTD-C (7CE0)	69.39 × (107/110) = 67.50	0.73391	2.476/107
CTD-D (7CE0)	68.93 × (107/110) = 67.05	0.73302	2.490/107

*L1 represents the length of the AlphaFold2 reference (Wuhan WT) model superimposed onto all other cryo-EM structures.

## Data Availability

The data presented in this study are openly available in ModelArchive under accession ma-gca-nprot (https://www.modelarchive.org/doi/10.5452/ma-gca-nprot) (released on 16 July 2024). Other data and information supporting the findings of this study are either public or available within this article and Supplementary Materials.

## Supplementary Materials

The following supporting information can be downloaded at https://www.mdpi.com/article/10.3390/v16091358/s1, Figure S1: Structural models of regions R1–R4 of high structural change showcasing the atomic-level differences for each VOC and haplotype; Figure S2: Normal mode analysis (NMA) of the N-protein; Table S1: Emergence of VOCs and haplotypes and their mutant constellations.
