# Supplementary material for: AlphaFold2 Reveals Structural Patterns of Seasonal Haplotype Diversification in SARS-CoV-2 Nucleocapsid Protein Variants
Source: Viruses. 2024 Aug 25;16(9):1358. doi: 10.3390/v16091358 (PMC11435742; doi:10.3390/v16091358)
Supplement: Supplementary file 1 [file viruses-16-01358-s001.zip › Figure S1.pdf]

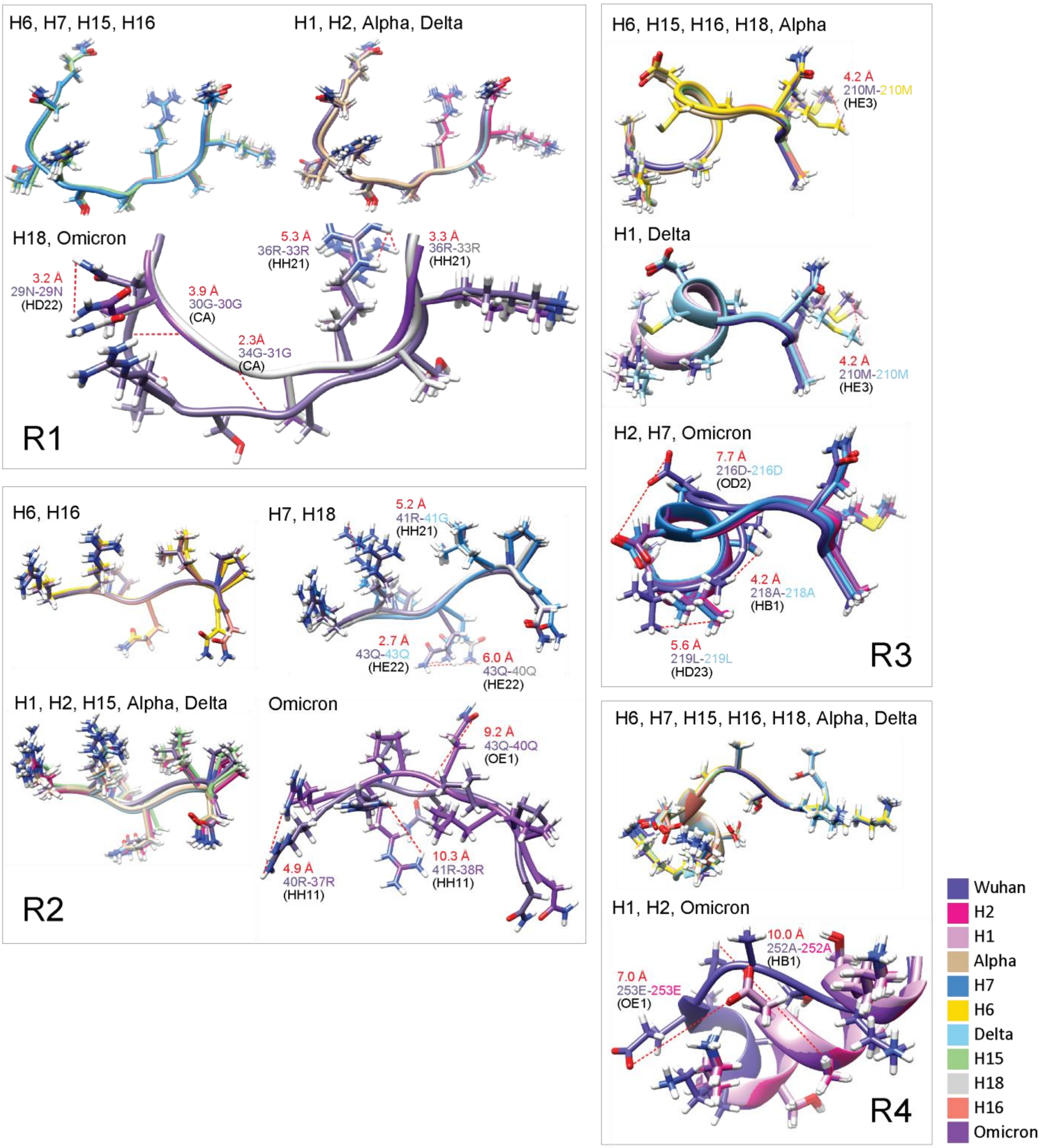

Figure S1. Structural models of regions R1-R4 of high structural change showcasing the atomic level differences for each VOC and haplotype. Distances between corresponding amino acids of the Wuhan reference against the VOC or haplotype are represented through dashed lines and measurements are in Ångstrom.
