# Supplementary material for: AlphaFold2 Reveals Structural Patterns of Seasonal Haplotype Diversification in SARS-CoV-2 Nucleocapsid Protein Variants
Source: Viruses. 2024 Aug 25;16(9):1358. doi: 10.3390/v16091358 (PMC11435742; doi:10.3390/v16091358)
Supplement: Supplementary file 1 [file viruses-16-01358-s001.zip › Figure S2.pdf]

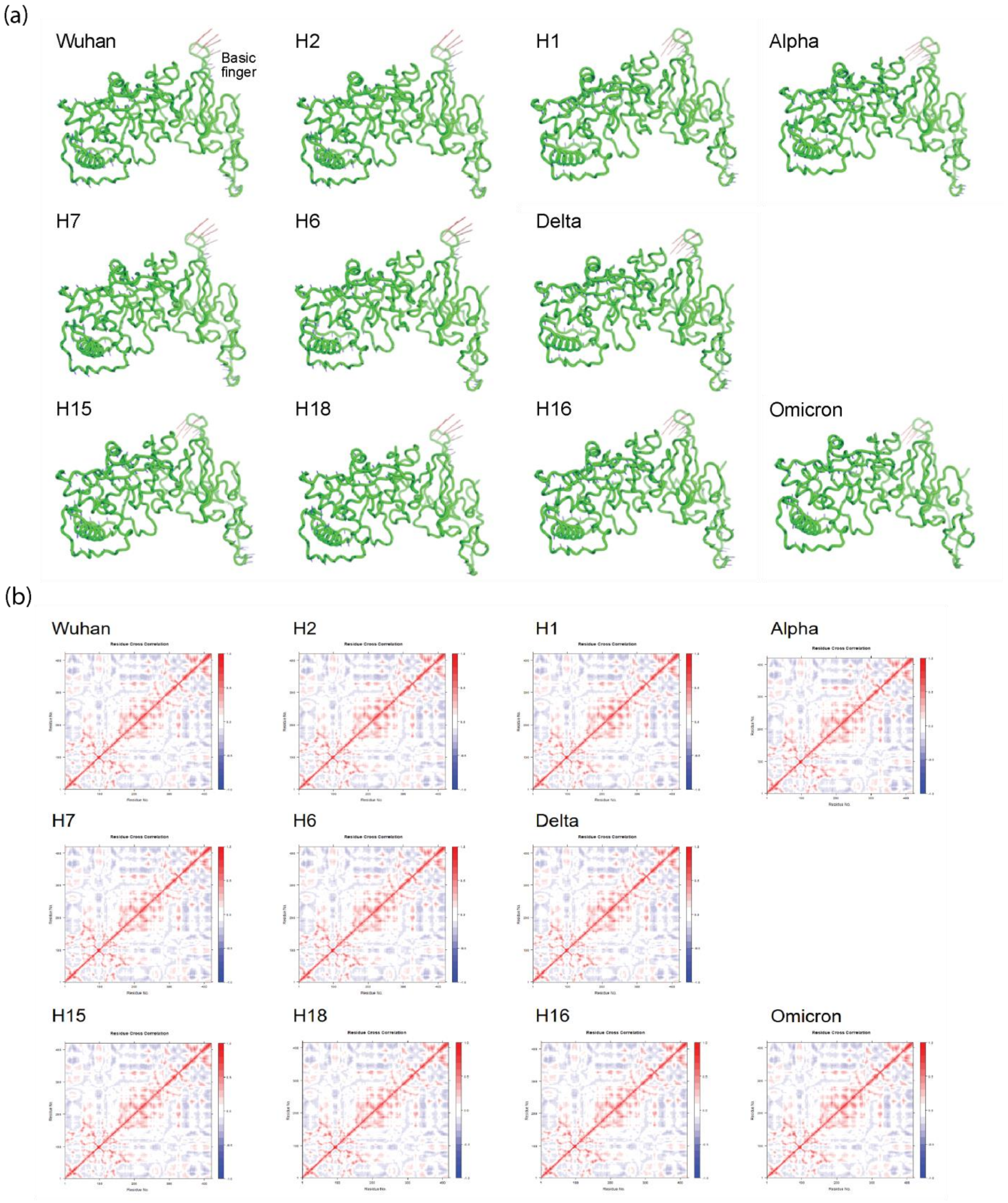

Figure S2. Normal mode analysis (NMA) of the N-protein. (a) Visualization of the first non-trivial mode (mode 7) for molecules of haplotypes and VOCs with vector field representations. (b) Cross-correlation heat maps averaged over all modes.
