# Supplementary material for: AlphaFold2 Reveals Structural Patterns of Seasonal Haplotype Diversification in SARS-CoV-2 Nucleocapsid Protein Variants
Source: Viruses. 2024 Aug 25;16(9):1358. doi: 10.3390/v16091358 (PMC11435742; doi:10.3390/v16091358)
Supplement: Supplementary file 1 [file viruses-16-01358-s001.zip › Table S1.pdf]

Table S1. Emergence of VOCs and haplotypes and their mutant constellations. Time of origin is given in a scale of relative appearance. Data from ref. [14].

Haplotype/VOC	Mutations	Time of Origin
**Omicron**	P13L, E31-, R32-, S33-, R203K, G204R, S413R	10
**H16**	S413R	9
**H18**	E31-, R32-, S33-	8
**H15**	P13L	7
**Delta**	D63G, R203M, G215C, D377Y	6
**H6**	G215C	5
**H7**	D63G, R203M, D377Y	4
**Alpha**	D3L, R203K, G204R, S235F	3
**H1**	D3L, S235F	2
**H2**	R203K, G204R	1
**Wuhan**	–	0
